# A Systematic Review and Meta-Analysis of the Effects of Herbal Medicine Buyang Huanwu Tang in Patients with Poststroke Fatigue

**DOI:** 10.1155/2021/4835488

**Published:** 2021-12-14

**Authors:** Chul Jin, Seungwon Kwon, Seung-Yeon Cho, Seong-Uk Park, Woo-Sang Jung, Sang-Kwan Moon, Jung-Mi Park, Chang-Nam Ko, Ki-Ho Cho

**Affiliations:** Department of Cardiology and Neurology, College of Korean Medicine, Kyung Hee University, Seoul, Republic of Korea

## Abstract

Poststroke fatigue (PSF) is reported to occur in 30%–72% of all patients with stroke. PSF not only is a symptom of stroke but has also been reported to adversely affect the prognosis of patients with stroke. However, no treatment has had a significant effect on PSF. In East Asian countries, several herbal medicines have been used to treat stroke, with Buyang Huanwu Tang (BHT) being the most common. This review aimed to evaluate the efficacy and safety of BHT for PSF. A literature search was conducted on MEDLINE, CENTRAL, Scopus, CiNii, CNKI, OASIS, NDSL, and KTKP databases for randomized controlled trials that evaluated the effects and safety of BHT on PSF. Six studies (*n* = 427) were included. The overall methodological quality of these studies was relatively low. In the adjunctive BHT group, the meta-analysis indicated statistically significant improvements in the Fatigue Severity Scale score (mean difference −1.49, 95% CI [−2.25, −0.73]) and total clinical efficacy rate (risk ratio 0.11, 95% CI [0.03, 0.41]) compared to those in the nonherbal group. Adverse events were only reported in one study, and no serious adverse events occurred. BHT administration might be effective in the treatment of PSF. We were unable to draw definitive conclusions owing to the limitations of the included studies. The trial is registered with CRD42019130178 in PROSPERO.

## 1. Introduction

Poststroke fatigue (PSF) can be defined as a feeling of early exhaustion with weariness, lack of energy, and aversion to effort that develops during physical or mental activity after a stroke that is usually not ameliorated by rest [1]. PSF has been reported to occur in 30%–72% of all stroke patients [2–5], with a potentially negatively effect on prognosis. In particular, PSF is known to adversely affect activities of daily living in patients with stroke [6]. As a result, patients with PSF show decreased participation in physical activity and rehabilitation treatment [6], resulting in poor neurological recovery, high mortality, and poor quality of life [7, 8]. Most of the recovery after a stroke has been reported to occur in the first 3–6 months after onset [9]. Therefore, patients who require rehabilitation treatment and feel significant fatigue need to be promptly assessed and treated.

Various pharmacological and nonpharmacological interventions have been used to treat PSF [10]. Specifically, fluoxetine and citicoline have been applied as pharmacological interventions, while fatigue education programs and mindfulness-based stress reduction programs have been attempted as nonpharmacological treatments [10]. However, a recent systematic review and meta-analysis suggested that there was insufficient evidence to support the use of any of these interventions to treat PSF [10]. The cause of PSF is multidimensional, including factors related to demographic data (e.g., age, sex, and marital status), neurological and physical deficits, medical comorbidities, smoking status, the use of certain medications, sleep disturbances, pain, pre-stroke fatigue, depression, anxiety, and cognitive impairment [11]. The pathophysiology of PSF is also unclear, with altered cortical excitability, inflammation, immune response, and genetic factors reported as major pathophysiological factors associated with PSF [11]. We hypothesize that these complex multidimensional influences on PSF are the main hindrances to the development of effective treatments.

Therefore, in this review, we focused on traditional East Asian herbal medicine. In East Asian countries, herbal medicine has been used to treat stroke. According to the results of a previous systematic review and meta-analysis [12], adjunctive treatment for acute cerebral infarction using herbal medicine is known to help alleviate neurological deficits. Various herbal medicines have been assessed in previous systematic reviews and meta-analyses, with Buyang Huanwu Tang (BHT, Boyang Hwano Tang in Korean, Hoyangkango in Japanese) and its variants being the most common among them [12]. BHT is a prescription that has been used for stroke patients with “Qi deficiency” and “blood stasis,” which is a type of pattern identification used in traditional East Asian medicine [13]. Since Qi deficiency is known to be closely related to fatigue symptoms [14, 15], we assumed that BHT could be effective not only for stroke itself but also for PSF.

Although previous studies have assessed the effects of BHT on stroke, no systematic literature review and meta-analysis has been conducted to evaluate the efficacy and safety of BHT for PSF. Therefore, in the present study, we systematically reviewed the clinical effect and safety of BHT on PSF based on the results of published randomized controlled trials (RCTs).

## 2. Methods

The trial registration number of this systematic review and meta-analysis was CRD42019130178 in PROSPERO. The present study was conducted based on a previously published protocol [16]. We followed the methods of Kwon et al., 2021 [17].

### 2.1. Database Search

Electronic databases including MEDLINE (via PubMed), the Cochrane Central Register of Controlled Trials (CENTRAL), Scopus, Citation Information by Nii (CiNii), China National Knowledge Infrastructure Database (CNKI), Oriental Medicine Advanced Searching Integrated System (OASIS), National Digital Science Library (NDSL), and Korean Traditional Knowledge Portal (KTKP) were searched up to October 2021. The search strategies were modified based on the characteristics of the individual databases; however, the main keywords used were as follows: “(Buyang Huanwu OR Boyang Hwano OR Hoyangkango) AND (Poststroke fatigue OR PSF OR stroke fatigue OR fatigue after stroke OR [cerebrovascular accident AND fatigue] OR [CVA AND fatigue] OR [stroke AND fatigue]),” which were appropriately combined for each database search. The specific search terms used for each database are presented in Appendix 1. There was no language or other publication restrictions in the study selection process.

### 2.2. Eligibility Criteria

The studies were screened and selected according to the inclusion criteria and subsequently reviewed. Only RCTs and quasi-RCTs investigating BHT for PSF treatment were included; nonrandomized clinical trials, case reports and series, uncontrolled trials, and laboratory (experimental) studies were excluded. Additionally, only patients with PSH who were diagnosed using a qualified clinical diagnostic method (such as the Fatigue Assessment Scale [FAS] and the Fatigue Severity Scale [FSS]) or subjective fatigue symptoms were included; patients with other conditions that could cause fatigue, such as cancer, chronic kidney disease, and liver cirrhosis, were excluded. There were no restrictions based on sex, ethnicity, symptom severity, disease duration, or clinical setting. Only studies that used BHT or modified (herbs added) BHT as experimental interventions were included. There were no limitations on dosage, frequency, duration of treatment, or formulation (e.g., decoctions, extracts, tablets, capsules, and powders). For the control group, only studies that included placebos, a no-treatment group, or conventional Western medicine therapies were included. Studies that used other traditional Chinese medicine therapies (including traditional Korean medicine and Japanese Kampo Medicine), such as those using different types of herbal medicines, acupuncture treatments, or moxibustion treatments, were excluded. Following the electronic search, two independent researchers (SK and CJ) screened and selected the studies according to the eligibility criteria. After any duplicates were removed, the remaining studies were assessed based on their titles and abstracts.

### 2.3. Outcome Assessment

The primary outcome was the FAS score. For secondary outcomes, we used other parameters such as the FSS score, total clinical efficacy rate (TCER, percentage of patients whose fatigue symptoms improved), and inflammatory cytokine levels. TCER was defined as the sum of the categories identified with the terms “cure,” “improved,” or “slightly improved.” In other words, all categories other than “unchanged” or “worse” were included in the TCER. The number and type of adverse events were also investigated.

### 2.4. Data Extraction and Quality Assessment of Individual Studies

**Figure 1:**
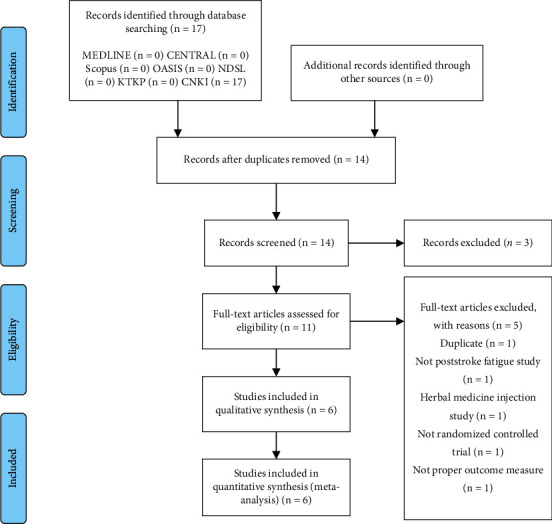
PRISMA flow diagram of study selection.

Two independent reviewers (SK and CJ) who had received training regarding the process and purpose of selection independently reviewed the titles, abstracts, and manuscripts of the studies to determine if they were eligible for inclusion in the analysis. All studies were uploaded to EndNote X7 (Clarivate Analytics). The reasons for excluding studies were recorded and are shown in a Preferred Reporting Items for Systematic Reviews and Meta-Analyses (PRISMA) flow chart (Figure 1). All disagreements were resolved through discussion between the two reviewers. Following this, two review writers (SK and CJ) independently extracted the data and completed a standard data extraction form, which included study information such as the first author, publication year, language, research design, sample size, characteristics of the participants (e.g., age and sex), details of randomization, blinding, interventions, treatment period, outcome measurements, primary outcomes, secondary outcomes, and statistical methods used. If there were disagreements, another review writer (WSJ) was consulted and assisted in the decision-making.

The quality and risk of bias assessment was performed based on the Cochrane Collaboration tool [18] and was conducted by two independent reviewers (SK and CJ). Any disagreements were resolved by an arbiter (WSJ) who made the final decision. A total of seven domains, including sequence generation, allocation concealment, blinding of participants, blinding of outcome assessors, incomplete outcomes, selective outcome reporting, and other risks of bias, were assessed. Each domain was evaluated as having a high risk of bias (H), low risk of bias (L), or unclear risk of bias (U).

### 2.5. Synthesis of Data and Meta-Analysis

Dichotomous data, such as the TCER, are presented using the risk ratio (RR) and 95% confidence interval (CI). Continuous data such as the FAS and FSS are presented as the mean difference (MD). A fixed-effect model was used for the meta-analysis when no statistical heterogeneity was found. The statistical heterogeneity of the risk factors among the trials was analyzed using *I*^2^. When *I*^2^ was <50%, it was assumed that no statistical heterogeneity was observed. In addition, the heterogeneity of the study methodologies was evaluated. In cases where the heterogeneity of one study was greater than that of the others, the relevant study was excluded from the analysis. For example, if the specific details of the intervention, such as the duration of treatment and the method used in one study, were clinically different from those of other studies, the study was excluded. The RevMan 5.3.5 software recommended by the Cochrane Collaboration (Oxford, UK) was used for all data analyses (http://tech.cochrane.org/revman/).

## 3. Result

### 3.1. Study Selection and Characteristics

Seventeen studies were retrieved by electronic search and included in the first screening stage. Of these, eleven studies were further assessed for eligibility to be included in the meta-analysis by a full reading of the text. Five studies were excluded for the following reasons: duplication (*n* = 1), ineligible participants (*n* = 1), ineligible interventions (*n* = 1), ineligible study design (*n* = 1), and ineligible outcome measurements (*n* = 1) (Figure 1). After reviewing the full text of each study, six studies (427 patients with PSF) [19–24] were finally included in the systematic review and meta-analysis (Table 1). All the six studies were written in Chinese and conducted in China [19–24].

There were two types of comparisons in the included studies: (1) herbal medicine + conventional therapies (including rehabilitation therapies for stroke) vs. conventional therapies alone (including rehabilitation therapies for stroke) (5 studies) [19, 20, 22–24] and (2) herbal medicine vs. conventional therapies (1 study) [21].

FAS was not evaluated in any of the included studies, TCER was evaluated in one study [20], and FSS was evaluated in five studies [19, 21–24]. The inflammatory cytokine levels of interleukin and tumor necrosis factor-*α* (TNF-*α*) were evaluated in one study [22].

The herbal medicines used in the studies were all BHT variants [19–24]. For each study, several herbs were added to the basic composition of BHT. Details regarding these compositions are shown in Table 2.

### 3.2. Risk of Bias within the Studies

**Figure 2:**
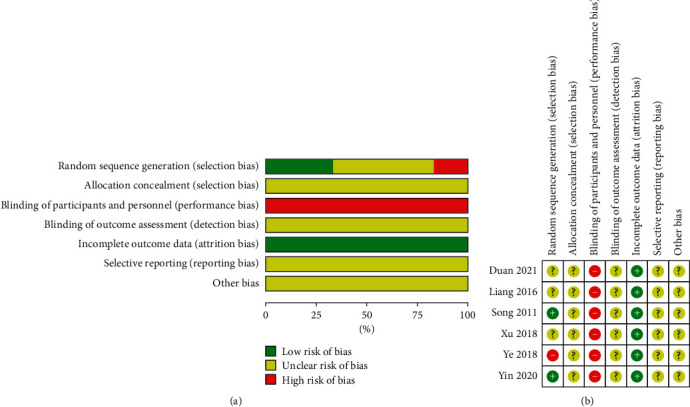
(a) Risk of bias graph: review of the authors' judgments regarding each risk of bias item is presented as percentages across all included studies. (b) Risk of bias summary: review of authors' judgments regarding each risk of bias item for each included study. “+”: low risk, “?”: unclear risk, and “−”: high risk.

In most studies, the risk of bias was high. Among the seven bias domains, concerns regarding random sequence generation, allocation concealment, blinding of participants, and outcome assessors were present. However, two studies had only low risk of bias in two items (randomization sequence generation and incomplete outcome data) [22, 23]. All studies [19–24] demonstrated a low risk of bias in incomplete outcome data. A summary of the risk of bias assessment is shown in Figure 2.

### 3.3. Fatigue Severity Scale

**Figure 3:**
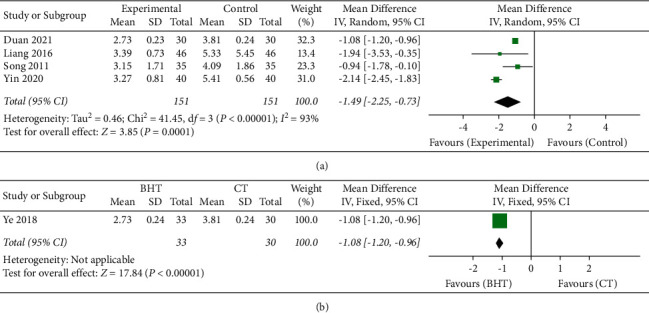
(a) FSS: Buyang Huanwu Tang + conventional therapies vs. conventional therapies only. (b) FSS: Buyang Huanwu Tang vs. conventional therapies BHT: Buyang Huanwu Tang; CT: conventional therapy; FSS: Fatigue Severity Scale.

Four of the studies [19, 22–24] that compared add-on BHT variants (conventional therapies including rehabilitation therapies for stroke + BHT variant) with a control treatment (conventional therapies including rehabilitation therapies for stroke only) assessed fatigue using the FSS. The meta-analysis showed a significantly lower FSS score in the BHT variant group than in the control group (MD −1.49, 95% CI [−2.25, −0.73]) (Figure 3(a)).

One RCT [21] compared the FSS scores in the BHT variant group with those in the conventional therapy group. In this RCT, the BHT variant group received only the BHT variant, while the control group received only conventional therapy. The BHT variant group showed significantly lower FSS scores than the control group (−1.08, 95% CI [−1.20, −0.96]) (Figure 3(b)).

### 3.4. Total Clinical Effective Rate

One of the RCTs [20] that compared add-on BHT variants (conventional therapies including rehabilitation therapies for stroke + BHT variant) with a control treatment (conventional therapies including rehabilitation therapies for stroke only) assessed TCER. In this RCT, the BHT variant group showed significantly lower RR than the control group (0.11, 95% CI [0.03, 0.41], *p*=0.002).

### 3.5. Inflammatory Cytokine Levels

Only one of the studies [22] that compared add-on BHT variants (conventional therapies including rehabilitation therapies for stroke + BHT variant) with a control treatment (conventional therapies including rehabilitation therapies for stroke only) assessed the inflammatory cytokine levels of interleukin (IL)-1*β*, IL-6, and TNF-*α*. Notably, TNF-*α* levels were significantly lower in the BHT variant group than in the control group (MD −9.20, 95% CI [−18.19, −0.21], *p*=0.011). However, the IL-1*β* and IL-6 levels were not significantly different between the two groups (IL-1*β*, MD −5.40, 95% CI [−11.44, 0.64]; IL-6, MD −5.50, 95% CI [−11.24, 0.24]).

### 3.6. Safety

Only one study [22] investigated adverse events, and no adverse effects were reported in either group in that study (Table 1).

## 4. Discussion

### 4.1. Main Findings

PSF is known to occur in 30%–72% of all stroke patients [2–5], and it has been reported to induce physical deconditioning, which results in reduced self-efficacy in terms of physical performance, poor rehabilitation participation and outcomes, reduced social participation, poor quality of life, functional limitations, and increased mortality [25]. Therefore, an appropriate treatment for PSF is crucial for improving outcomes. However, sufficient evidence for the treatment or prevention of PSF has not been found for several pharmacological agents (e.g., antidepressants) and nonpharmacological approaches (e.g., mindfulness-based stress reduction and fatigue education programs) [10]. Therefore, finding alternative treatment options is essential. The results of the meta-analysis in the present study indicate that the administration of herbal medicine (BHT or BHT variants) might result in significant improvements in PSF in stroke patients. The administration of BHT or BHT variants for PSF was found to significantly reduce the FSS scores and TCER.

### 4.2. The Mechanism of BHT or BHT Variants on PSF

BHT is known to treat the condition called “blood stasis” in traditional East Asian medicine and is used for the treatment of hemiparesis in patients with stroke [13, 26]. The herbal prescriptions used to treat blood stasis in traditional East Asian medicine have varied. While BHT is one of the treatment options, it is different from other herbal prescriptions for blood stasis treatment in that it has mainly been used for blood stasis with Qi deficiency [13]. Qi deficiency, a type of pattern identification that has been used in clinical practice in traditional East Asian medicine, is known to be closely associated with fatigue symptoms. A previous study has suggested that Qi deficiency is associated with fatigue in patients with breast cancer [14]. In addition, according to the standardized predictive models for traditional Korean medical diagnostic pattern identification in stroke subjects previously developed by this study team, the likelihood of a Qi deficiency diagnosis was significantly increased when a patient had symptoms such as pale complexion, faint low voice, reversal cold of the extremities, and fatigue [15]. Therefore, we assumed that BHT, which has been used for Qi deficiency in stroke patients, would be effective for PSF.

This application could also be explained by the pharmacological mechanism of action of BHT. A previous experimental study has suggested that BHT has a neuroprotective effect in cerebral ischemic conditions [27]. The administration of BHT to mice with middle cerebral ischemic/reperfusion injury has been shown to lead to a significant downregulation of the genes involved in inflammation, apoptosis, angiogenesis, and blood coagulation as well as the upregulation of the genes mediating neurogenesis and nervous system development [27]. While the number of studies is not substantial, a link between stroke-induced inflammation and PSF has been suggested in some studies [28]. Therefore, BHT could be effective against PSF through the inhibition of stroke-induced inflammation.

### 4.3. Strengths and Limitations

This study has a number of strengths. First, this is the first systematic review and meta-analysis to evaluate the effects of BHT or its variants on PSF. Second, no restriction on language was applied during the literature search process. Therefore, we were able to review all the relevant studies available. Furthermore, we provided a detailed summary of the BHT or BHT variant compositions used in the studies (Table 2). This information can be utilized by clinicians prescribing BHT to patients with PSF in clinical settings.

However, there are some limitations to the present study. First, all eligible studies exhibited methodological flaws, such as in terms of patient selection, performance, and detection bias. Only two studies had low risk of bias in two items (randomization sequence generation and incomplete outcome data) [22, 23]. Second, detailed information regarding the protocols used in the RCTs was not provided in the included studies. Third, all of the eligible studies, except one [22], did not report adverse events. Fourth, most included studies had different BHT and BHT variant components (Table 2) [19–24]. There are five types of BHT and their variants. Although Astragali Radix, Angelicae Gigantis Radix, Cnidii Rhizoma, Paeoniae Radix Rubra, Lumbricus, Carthami Flos, and Persicae Semen were commonly present in all types of BHT, the specific dosages were all different. In addition, the three types of BHT contain various herbs, in addition to the common herbs mentioned above [19, 22, 23]. Fifth, only 17 studies were found using an electronic search, and only 6 of them could be analyzed. As a result, both the quantity and quality of evidence are insufficient to reach a concrete conclusion. Lastly, all the trials included in this study were conducted in China [19–24]. Therefore, there could be a publication bias given the high rate of studies published in China. A previous study has called attention to the exceptionally high success rate of trials conducted in China compared to those conducted in other regions [29]. Therefore, it is possible that the results of this meta-analysis reflect only the clinical settings in China and may not be applicable to patients in other countries or of other ethnic backgrounds (besides North East Asia). Based on these limitations, we could not draw concrete conclusions from the current literature.

### 4.4. Comparison with Existing Literature

The present study is the first systematic review and meta-analysis to evaluate the efficacy of the alternative therapeutic options of BHT and BHT variants on PSF. However, several studies have been conducted on conventional pharmacological and nonpharmacological treatments for PSF. Based on these studies, a Cochrane review was first published in 2009 to evaluate the efficacy of pharmacological and nonpharmacological interventions on PSF [30], and an updated version was published in 2015 [10]. However, both studies failed to find significant improvements using either pharmacological approaches, such as the antidepressant fluoxetine, or nonpharmacological approaches, such as education programs.

The present systematic review showed that the administration of BHT and BHT variants might be effective in treating PSF. Additionally, the results of this study showed that BHT could be a new therapeutic option for patients with PSF. However, it is difficult to draw definitive conclusions regarding the efficacy and safety of BHT owing to the limitations of the included studies.

### 4.5. Implications for Research

First, to provide a higher level of evidence, the heterogeneity of the interventions used in future studies should be reduced. Specific details regarding the BHT compositions used in the eligible RCTs were all different. Therefore, it is necessary to standardize the composition of BHT for future clinical trials. Second, the risk of bias should be minimized as much as possible. Above all, specific information about allocation concealment and blinding of participants, investigators, and statisticians should be provided. It is also essential to use a placebo in the control group. If the patients, evaluators, and statisticians are not blinded, this could influence the results. However, making a placebo for herbal medicines tends to be difficult due to their unique color, taste, and flavor [31]. Therefore, establishing a process for making an effective placebo should be the focus of future research. Finally, future studies should provide detailed information regarding their study protocols, such as protocol registration numbers.

## 5. Conclusions

In conclusion, we could suggest the administration of BHT and BHT variants to treat patients with PSF might reduce their clinical symptoms and inflammatory cytokine levels. However, the quality of the studies reviewed was generally low, and there was insufficient data to draw concrete conclusions regarding the efficacy and safety of BHT and BHT variants in patients with PSF. Therefore, further studies are required to confirm these findings.

## Figures and Tables

**Table 1:** Characteristics of the included studies.

Study (first author, year)	Subjects (herbal/control)	Stroke type (ischemic/hemorrhagic)	Interventions		Main outcomes (herbal/control)	Duration of outcome assessment (weeks)	Adverse events (herbal/control)
			Herbal medicines	Control group			
Song, 2011	70 (35/35)	53/17	Conventional therapies + rehabilitation therapies for stroke + BHT variant 1 4 weeks	Conventional therapies + rehabilitation therapies for stroke 4 weeks	FSS 3.15 ± 1.71/4.09 ± 1.86 IL-1*β* (23/19) 21.3 ± 9.5/26.7 ± 10.3 (pg/ml) IL-6 (23/19) 16.2 ± 8.3/21.7 ± 9.2 (pg/ml) TNF-*α* (23/19) 20.6 ± 14.3/29.8 ± 15.2 (pg/ml)	4	0/0
Liang, 2016	92 (46/46)	57/35	Conventional therapies + rehabilitation therapies for stroke + BHT variant 2 4 weeks	Conventional therapies + rehabilitation therapies for stroke 4 weeks	FSS 3.39 ± 0.73/5.33 ± 5.45	4	Unreported
Xu, 2018	62 (31/31)	Unreported	Conventional therapies + rehabilitation therapies for stroke + BHT variant 3 4 weeks	Conventional therapies + rehabilitation therapies for stroke 4 weeks	TCER 29/31 (93.55%)/19/31 (61.29%)	4	Unreported
Yin, 2020	80 (40/40)	Unreported	Conventional therapies + rehabilitation therapies for stroke + BHT variant 4 4 weeks	Conventional therapies + rehabilitation therapies for stroke 4 weeks	FSS 3.27 ± 0.81/5.41 ± 0.56	4	Unreported
Duan, 2021	60 (30/30)	Unreported	Conventional therapies + rehabilitation therapies for stroke + BHT variant 5 4 weeks	Conventional therapies + rehabilitation therapies for stroke 4 weeks	FSS 2.73 ± 0.23/3.81 ± 0.24	4	Unreported
Ye, 2018	63 (33/30)	Unreported	BHT variant 5 4 weeks	Conventional therapies for stroke 4 weeks	FSS 2.73 ± 0.24/3.81 ± 0.24	4	Unreported

BHT: Buyang Huanwu Tang; FSS: Fatigue Severity Scale; TCER: total clinical efficacy rate.

**Table 2:** Herbal medicines and treatment details.

Study (first author, year, type of BHT)	Components of the herbal medicines (g/day)
Song, 2011, BHT variant 1	Astragali radix 60∼120 g, Angelicae gigantis radix 10 g, Cnidii rhizoma 15 g, paeoniae radix rubra 15 g, lumbricus 10∼15 g, Carthami flos 5 g, persicae semen 10 g, Achyranthis radix 15 g, Atractylodis rhizoma Alba 20 g, Cistanchis herba 20 g, Codonopsis pilosulae radix 15 g, epimedii herba 15 g, glycyrrhizae radix et rhizoma 5 g, pinelliae tuber 10 g, spatholobi Caulis 30 g
Liang, 2016, BHT variant 2	Astragali radix 50 g, Angelicae gigantis radix 10 g, Cnidii rhizoma 10 g, paeoniae radix rubra 15 g, lumbricus 10 g, Carthami flos 10 g, persicae semen 10 g, Atractylodis rhizoma Alba 10 g, bupleuri radix 10 g, glycyrrhizae radix et rhizoma 6 g, menthae herba 3 g, paeoniae radix Alba 10 g, poria sclerotium 10 g, zingiberis rhizoma recens 3 g
Xu, 2018, BHT variant 3	Astragali radix 120 g, Angelicae gigantis radix 12 g, Cnidii rhizoma 10 g, paeoniae radix rubra 15 g, lumbricus 10 g, Carthami flos 10 g, persicae semen 10 g
Yin, 2020 BHT variant 4	Astragali radix 25 g, Angelicae gigantis radix 25 g, Cnidii rhizoma 15 g, paeoniae radix Alba 10 g, lumbricus 10 g, Carthami flos 10 g, persicae semen 10 g, bupleuri radix 15 g, Citri unshius pericarpium 10 g, Atractylodis rhizoma Alba 10 g, poria sclerotium 20 g, Cyperi rhizoma 6 g, scorpio 3 g, zingiberis rhizoma recens 3 pieces
Duan, 2021 Ye, 2018, BHT variant 5	Astragali radix 120 g, Angelicae gigantis radix 10 g, Cnidii rhizoma 10 g, paeoniae radix rubra 15 g, lumbricus 10 g, Carthami flos 10 g, persicae semen 10 g

BHT: Buyang Huanwu Tang.

## Data Availability

The data used to support the findings of this study are included within the article.

## Supplementary Materials

Supplementary Materials Appendix 1: specific search terms used for each database.

## References

[1] Staub F., Bogousslavsky J. (2001). Fatigue after stroke: a major but neglected issue. *Cerebrovascular Diseases*.

[2] Feigin V. L., Barker-Collo S., Parag V. (2012). Prevalence and predictors of 6-month fatigue in patients with ischemic stroke: a population-based stroke incidence study in Auckland, New Zealand, 2002-2003. *Stroke*.

[3] Vuletić V., Lezaić Z., Morović S. (2011). Post-stroke fatigue. *Acta Clinica Croatica*.

[4] Colle F., Bonan I., Gellez Leman M.-C., Bradai N., Yelnik A. (2006). Fatigue après accident vasculaire cérébral. *Annales de Readaptation et de Medecine Physique*.

[5] Choi-Kwon S., Kim J. S. (2011). Poststroke fatigue: an emerging, critical issue in stroke medicine. *International Journal of Stroke*.

[6] Choi-Kwon S., Han S. W., Kwon S. U., Kim J. S. (2005). Poststroke fatigue: characteristics and related factors. *Cerebrovascular Diseases*.

[7] Glader E.-L., Stegmayr B., Asplund K. (2002). Poststroke fatigue. *Stroke*.

[8] Naess H., Lunde L., Brogger J. (2012). The effects of fatigue, pain, and depression on quality of life in ischemic stroke patients: the Bergen stroke study. *Vascular Health and Risk Management*.

[9] Skilbeck C. E., Wade D. T., Hewer R. L., Wood V. A. (1983). Recovery after stroke. *Journal of Neurology, Neurosurgery & Psychiatry*.

[10] Wu S., Kutlubaev M. A., Chun H. Y. (2015). Interventions for post-stroke fatigue. *Cochrane Database of Systematic Reviews*.

[11] Hinkle J. L., Becker K. J., Kim J. S. (2017). Poststroke fatigue: emerging evidence and approaches to management: a scientific statement for healthcare professionals from the American heart association. *Stroke*.

[12] Han C.-h., Kim M., Cho S.-Y. (2018). Adjunctive herbal medicine treatment for patients with acute ischemic stroke: a systematic review and meta-analysis. *Complementary Therapies in Clinical Practice*.

[13] Wei R. L., Teng H. J., Yin B. (2013). A systematic review and meta-analysis of buyang huanwu decoction in animal model of focal cerebral ischemia. *Evidence-based Complementary and Alternative Medicine: eCAM*.

[14] Chien T.-J., Song Y.-L., Lin C.-P., Hsu C.-H. (2012). The correlation of traditional Chinese medicine deficiency syndromes, cancer related fatigue, and quality of life in breast cancer patients. *Journal of Traditional and Complementary Medicine*.

[15] Jung W.-S., Cho S.-Y., Park S.-U. (2019). Development of standardized predictive models for traditional Korean medical diagnostic pattern identification in stroke subjects: a hospital-based multi-center trial. *Journal of Korean Medicine*.

[16] Jin C., Cho S.-Y., Park S.-U. (2019). Buyang huanwu Tang (Boyang Hwano Tang) for the treatment of post-stroke fatigue. *Medicine (Baltimore)*.

[17] Kwon S., Jin C., Chung M. (2021). Herbal medicine treatment for patients with chronic subdural hematoma: a systematic review and meta-analysis. *Complementary Therapies in Clinical Practice*.

[18] Higgins J. P. T., Altman D. G., Gotzsche P. C. (2011). The cochrane collaboration’s tool for assessing risk of bias in randomised trials. *BMJ*.

[19] Liang Y., Gong W., Su Y., Li C. (2016). Observation on the effect of buyang huanwu decoction and xiaoyao powder in adjuvant treatment of post-stroke fatigue. *People Military Surgery*.

[20] Xu Y. (2018). Clinical observation on modified buyang huanwu decoction in the treatment of fatigue after stroke. *Journal of Practical Traditional Chinese Medicine*.

[21] Ye C., Chen Z., Wang L. (2018). Application of Buyang Huanwu decoction in the treatment of post-stroke fatigue patients in the community. *China Modern Doctor*.

[22] Song X. J. (2011). *Effect of the Combination of Jiawei Buyang Huanwu Decoction and Rehabilitation Training on the Serum Proinflammatory Cytokines in Patients with Post Stroke Fatigue*.

[23] Yin Y. (2020). Clinical observation of chaihu shugan powder and buyang huanwu decoction combined with western medicine in treating fatigue after stroke. *China’s Naturopathy.*.

[24] Duan D. (2021). Clinical analysis of buyang huanwu decoction in treating fatigue patients after stroke in community. *Journal of Mathematical Medicine*.

[25] Nadarajah M., Goh H.-T. (2015). Post-stroke fatigue: a review on prevalence, correlates, measurement, and management. *Topics in Stroke Rehabilitation*.

[26] Li H.-Q., Wei J.-J., Xia W. (2015). Promoting blood circulation for removing blood stasis therapy for acute intracerebral hemorrhage: a systematic review and meta-analysis. *Acta Pharmacologica Sinica*.

[27] Wang H.-W., Liou K.-T., Wang Y.-H. (2011). Deciphering the neuroprotective mechanisms of Bu-yang Huan-Wu decoction by an integrative neurofunctional and genomic approach in ischemic stroke mice. *Journal of Ethnopharmacology*.

[28] Wen H., Weymann K. B., Wood L., Wang Q. M. (2018). Inflammatory signaling in post-stroke fatigue and depression. *European Neurology*.

[29] Vickers A., Goyal N., Harland R., Rees R. (1998). Do certain countries produce only positive results? a systematic review of controlled trials. *Controlled Clinical Trials*.

[30] McGeough E., Pollock A., Smith L. N. (2009). Interventions for post-stroke fatigue. *Cochrane Database of Systematic Reviews*.

[31] Zhang X., Tian R., Zhao C., Tang X., Lu A., Bian Z. (2019). Placebo design in WHO-registered trials of Chinese herbal medicine need improvements. *BMC Complementary and Alternative Medicine*.

