# Supplementary material for: A Systematic Review and Meta-Analysis of the Effects of Herbal Medicine Buyang Huanwu Tang in Patients with Poststroke Fatigue
Source: Evid Based Complement Alternat Med. 2021 Dec 14;2021:4835488. doi: 10.1155/2021/4835488 (PMC8691996; doi:10.1155/2021/4835488)
Supplement: Supplementary Materials — Appendix 1: specific search terms used for each database. [file 4835488.f1.docx]

## Appendix

**Appendix 1.** Specific search terms used for each database

**MEDLINE**

| Search | Query |
| --- | --- |
| #1 | Search "poststroke fatigue"[tiab] OR "PSF"[tiab] OR "stroke fatigue"[tiab] OR "fatigue after stroke"[tiab] |
| #2 | Search "cerebrovascular accident"[tiab] AND "fatigue"[tiab] |
| #3 | Search "CVA"[tiab] AND "fatigue"[tiab] |
| #4 | Search "stroke"[tiab] AND "fatigue"[tiab] |
| #5 | #1 OR #2 OR #3 OR #4 |
| #6 | Search ("Buyang Huanwu Tang"[tiab] OR "Buyang Huanwu Decoction"[tiab] OR "Buyang Huanwu"[tiab]) OR ("Boyang Hwano Tang"[tiab] OR "Boyang Hwano Decoction"[tiab] OR "Boyang Hwano "[tiab]) OR ("Hoyangkangoto "[tiab] OR "Hoyangkango"[tiab] OR " Hoyangkango*"[tiab]) |
| #7 | Search "randomise*"[tiab] OR "randomize*"[tiab] |
| #6 | #5 AND #6 AND #7 Filters: Clinical Trial |

**CENTRAL**

| ID | Search |
| --- | --- |
| #1  #2  #3  #4  #5 | 'poststroke fatigue' OR 'PSF' OR 'stroke fatigue':ti,ab,kw (Word variations have been searched)  'cerebrovascular accident ' AND 'fatigue':ti,ab,kw (Word variations have been searched)  'CVA' AND 'fatigue':ti,ab,kw (Word variations have been searched)  'stroke' AND 'fatigue':ti,ab,kw (Word variations have been searched)  #1 or #2 or #3 or #4 |
| #6 | 'Buyang Huanwu' OR 'Boyang Hwano' OR 'Hoyangkango':ti,ab,kw (Word variations have been searched) |
| #7 | 'randomized' OR 'randomised':ti,ab,kw (Word variations have been searched) |
| #8 | #5 and #6 and #7 |

**SCOPUS**

#1 “poststroke fatigue” OR “PSF” OR “stroke fatigue” [TITLE-ABS-KEY]

#2 “cerebrovascular accident” AND “fatigue” [TITLE-ABS-KEY]

#3 “CVA” AND “fatigue” [TITLE-ABS-KEY]

#4 “stroke” AND “fatigue” [TITLE-ABS-KEY]

#5 #1 OR #2 OR #3 OR #4

#6 “Buyang Huanwu” OR “Boyang Hwano” OR “Hoyangkango”

#7 “randomized” OR “randomised”

#8 #5 AND #6 AND #7

**KTKP, NDSL, OASIS**

보양환오 or 뇌졸중 후 피로

**CNKI**

#1 脑卒中 OR 中风

#2 疲劳

#3 #1 AND #2

#4 补阳还五

#5 随机

#6 AND / #3-#5

**CiNii**

Hoyangkango AND Stroke AND Fatigue
